# The Effect of Nitrogen Annealing on the Resistive Switching Characteristics of the W/TiO_2_/FTO Memory Device

**DOI:** 10.3390/s23073480

**Published:** 2023-03-27

**Authors:** Zhiqiang Yu, Xu Han, Jiamin Xu, Cheng Chen, Xinru Qu, Baosheng Liu, Zijun Sun, Tangyou Sun

**Affiliations:** 1 Faculty of Electronic Engineering, Guangxi University of Science and Technology, Liuzhou 545006, China; 2 Wuhan National Laboratory for Optoelectronics, School of Optical and Electronic Information, Huazhong University of Science and Technology, Wuhan 430074, China; 3 Guangxi Key Laboratory of Precision Navigation Technology and Application, Guilin University of Electronic Technology, Guilin 541004, China

**Keywords:** TiO_2_ nanowire, memory device, nonvolatile, oxygen vacancies

## Abstract

In this paper, the effect of nitrogen annealing on the resistive switching characteristics of the rutile TiO_2_ nanowire-based W/TiO_2_/FTO memory device is analyzed. The W/TiO_2_/FTO memory device exhibits a nonvolatile bipolar resistive switching behavior with a high resistance ratio (R_HRS_/R_LRS_) of about two orders of magnitude. The conduction behaviors of the W/TiO_2_/FTO memory device are attributed to the Ohmic conduction mechanism and the Schottky emission in the low resistance state and the high resistance state, respectively. Furthermore, the R_HRS_/R_LRS_ of the W/TiO_2_/FTO memory device is obviously increased from about two orders of magnitude to three orders of magnitude after the rapid nitrogen annealing treatment. In addition, the change in the W/TiO_2_ Schottky barrier depletion layer thickness and barrier height modified by the oxygen vacancies at the W/TiO_2_ interface is suggested to be responsible for the resistive switching characteristics of the W/TiO_2_/FTO memory device. This work demonstrates the potential applications of the rutile TiO_2_ nanowire-based W/TiO_2_/FTO memory device for high-density data storage in nonvolatile memory devices.

## 1. Introduction

The memristor has been defined as the fourth circuit element after the capacitor, resistor, and inductor. In 1971, Chua [1] had already guessed that there should be a circuit element that could convert the magnetic flux and charge to each other according to the relationship between the four variables of the circuit, deduced that the device could memorize the characteristics of the resistance, and named it a memristor. In 2008, HP Labs [2] produced the first nanoscale memristor, which attracted the extensive attention of a large number of researchers due to the unique properties of the memristor itself. Up to now, memristors have been widely used in artificial neural networks and synapses [3,4,5], chaotic circuits [6], and secure communications [7]. Sun et al. [8] designed a neural network circuit that could relate emotion and memory based on the memristor circuit. Yang et al. [9] embedded graphene–oxide quantum dots in an HfO_2_ memristor, which showed low charge loss while ensuring long-term stability, demonstrating the potential application in the nonvolatile memory devices. It is worth noting that the traditional storage technologies have encountered development bottlenecks, and the storage volume of the traditional memory devices is about to reach the size limit according to Moore’s Law [10,11,12]. Thus, it is urgent to develop a new type of memory device to overcome the development bottlenecks of the traditional memory devices, and nanoscale memristors may be a potential candidate for application in the future nonvolatile memory devices.

A memristor is composed of a metal–semiconductor–metal MIM structure. The choice of the intermediate semiconductor layer for the memristor has a crucial influence on the resistive switching characteristics of the memristor. The transition-metal–oxide-based memristors have shown many excellent advantages such as an easy fabrication process, good compatibility, and simple compositions [13]. Among them, the transition metal oxide TiO_2_ is widely used in photocatalysis [14] and sensors [15] due to its low cost, easy fabrication, and corrosion resistance. Moreover, another important factor affecting the performances of the memristors is the preparation process. The transition metal oxide TiO_2_ has been prepared by the hydrothermal method [16,17,18,19,20], the magnetron sputtering method [21,22], the electrochemical anodization method [23,24], the atomic layer deposition method (ALD) [25,26], and other methods [27,28,29]. In particular, the hydrothermal method with its simple experimental steps and high economic benefits is an effective approach to prepare the transition metal oxide TiO_2_. Recently, various preparation methods have been developed to prepare TiO_2_ memristors. Zhang et al. [17] prepared rutile TiO_2_ nanorods by the hydrothermal method, and the resistance ratio of the TiO_2_ nanorod-based Pt/TiO_2_ NRAs/FTO device was about 10. Wang et al. [21] carried out a study on the resistive switching property of the amorphous TiO_2_ thin film deposited by the radio frequency magnetron sputtering method on a flexible copper (Cu) foil substrate, and the resistance ratio of the TiO_2_ thin film-based Ni/TiO_2_/Cu device was almost one magnitude. Chen et al. [23] fabricated the Au/Cu NWs/TiO_2_ NTAs/Ti device with a resistance ratio greater than 40 by the electrochemical anodization method.

The one-dimensional TiO_2_ nanowires have recently received great interest for different nanoelectronics and optoelectronics applications owning to their unique physical and chemical behaviors [16,30]. Furthermore, the enhanced resistive switching properties of the TiO_2_ memristors have been obtained by nitrogen (N_2_) annealing treatment [18,31]. However, the effect of nitrogen annealing on the resistive switching behavior and mechanism of the W/TiO_2_/FTO memory device has not been reported so far. Herein, the rutile TiO_2_ nanowire-based W/TiO_2_/FTO memory device was prepared, and the effect of the nitrogen annealing on the nonvolatile resistive switching behavior and mechanism of the W/TiO_2_/FTO memory device is reported.

## 2. Experiments

All the chemicals, including titanium butoxide (97%) and concentrated hydrochloric acid (36%–38% by mass), were of analytical grade and used without further purifying; they were purchased from Sigma-Aldrich. Fluorine-doped tin oxide (FTO, 15 Ω/square) was used as the substrate for the epitaxial growth of the TiO_2_ nanowire arrays. The preparation process was as follows: First, 15 mL of concentrated hydrochloric acid was mixed with 15 mL of deionized water and stirred continuously for 15 min at room temperature. Then, 0.5 mL of titanium butoxide was slowly dripped into the above mixed solution containing 15 mL of deionized water and 15 mL of concentrated hydrochloric acid. After sufficient stirring for another 15 min, a transparent solution was generated, which acted as the TiO_2_ precursor solution. Subsequently, the precursor solution was transferred into a 50 mL Teflon-lined stainless-steel autoclave, in which a piece of FTO substrate with the conducting surface facing down was kept at an angle against the inner wall of the Teflon liner. After that, the autoclave was sealed and continuously heated at 140 °C for 4 h. Finally, the Teflon-lined stainless-steel autoclave was cooled down to room temperature and the sample was taken out, washed with ethanol and deionized water, and then allowed to dry in ambient air. After the synthesis, the TiO_2_ sample was annealed at 450 °C in a tube furnace for 1 h under a N_2_ atmosphere. The tungsten electrodes were deposited on the TiO_2_ sample by the DC magnetron sputtering process.

The crystal structure and surface topography of the prepared samples were detected by X-ray diffraction (XRD) and a field emission scanning electron microscope (FESEM), respectively. X-ray Photoelectron Spectroscopy (XPS) was performed to survey the chemical composition and surface states of the TiO_2_ nanowire arrays. The I–V characteristics of the TiO_2_ nanowire-based W/TiO_2_/FTO memory device were measured by using an Agilent B2901A analyzer at room temperature.

## 3. Results and Discussion

### 3.1. The Structure and Morphology of the TiO_2_ Nanowire Arrays

**Figure 1:**
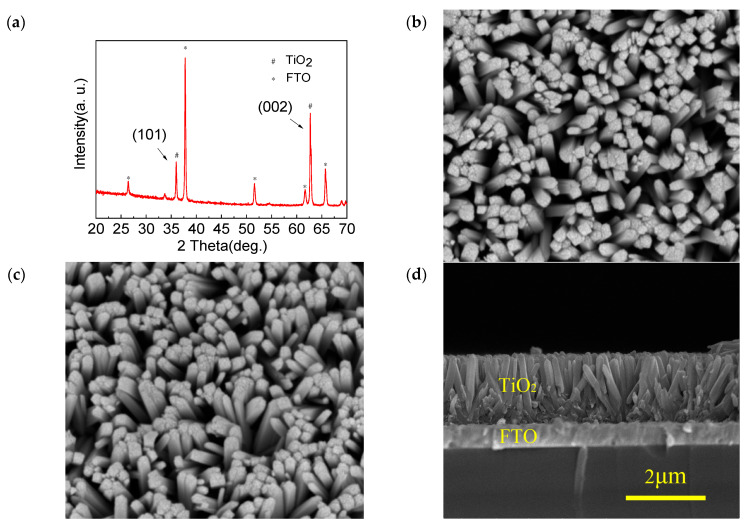
(**a**) XRD pattern of the TiO_2_ nanowire arrays. (**b**) Top-view FESEM image of the TiO_2_ nanowire arrays. (**c**) Tilt-view FESEM image of the TiO_2_ nanowire arrays. (**d**) Cross-sectional FESEM image of the TiO_2_ nanowire arrays.

Figure 1a shows the XRD pattern of the TiO_2_ nanowire arrays. It is clear that there were only two sharp diffraction peaks, such as (101) and (002), observed at 36.20° and 62.84°, respectively, which can be assigned to the tetragonal rutile phase (JCPDS No. 88-1175) [17]. Moreover, the (002) diffraction peak was clearly enhanced compared with the (101) diffraction peak, while some diffraction peaks such as the (111), (211), and (110) crystal planes were absent, which suggests that the highly oriented TiO_2_ nanowire grew preferentially along the [001] orientation with the growth axis perpendicular to the FTO substrate. Figure 1b,c indicate the top-view and tilt-view FESEM images of the TiO_2_ nanowire arrays, respectively. It is observed that the FTO substrate was densely and uniformly covered by the vertically aligned TiO_2_ nanowire arrays with smooth edges and rough top surfaces. Figure 1d displays the cross-sectional FESEM image of the TiO_2_ nanowire arrays. It is clearly shown that the average height and diameter of the rutile TiO_2_ nanowire arrays were about 1.5 μm and 180 nm, respectively.

### 3.2. The Chemical Composition and Surface States of the TiO_2_ Nanowire Arrays

**Figure 2:**
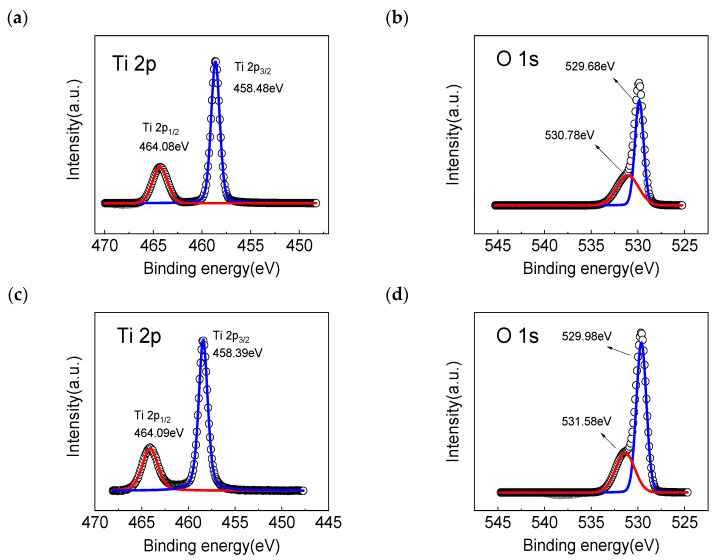
(**a**) Ti 2p and (**b**) O 1 s high resolution XPS spectra of the TiO_2_ nanowire arrays. (**c**) Ti 2p and (**d**) O 1 s high resolution XPS spectra of the TiO_2_ nanowire arrays after the rapid nitrogen annealing treatment.

Figure 2 shows the XPS spectra of the Ti 2p and O 1 s in the rutile TiO_2_ nanowire arrays before and after the rapid nitrogen annealing treatment. As shown in Figure 2a, the Ti 2p_3/2_ and Ti 2p_1/2_ peaks around 458.48 eV and 464.08 eV can be observed. The spin-orbit splitting binding energy between Ti 2p_3/2_ and Ti 2p_1/2_ was approximately 5.6 eV, indicating the existence of Ti-O bonds in the rutile TiO_2_ nanowire arrays. Figure 2b reveals the XPS spectrum of O 1 s. It was found that the peak at the binding energy of 529.68 should be attributed to the lattice oxygen in the rutile TiO_2_ nanowire arrays, while the peak at the binding energy of 530.78 eV may be assigned to the oxygen vacancies, which suggests that the as-prepared rutile TiO_2_ nanowire arrays contained a considerable amount of oxygen vacancies, after the rapid nitrogen annealing treatment at 450 °C for 1 h, as shown in Figure 2c,d. It is obvious that the Ti 2p_3/2_ and Ti 2p_1/2_ peaks were found at 458.39 eV and 464.09 eV, respectively. In addition, the peak area of the 531.58 eV binding energy corresponding to the oxygen vacancies in Figure 2c was about 12.4% larger than that of the 530.78 eV binding energy in Figure 2a, which means that the nitrogen atoms entered the rutile TiO_2_ nanowire arrays and replaced the lattice oxygen atoms [32], resulting in an increase in the oxygen vacancies in the rutile TiO_2_ nanowire arrays.

### 3.3. The Electrical Characteristics of the W/TiO_2_/FTO Memory Device

**Figure 3:**
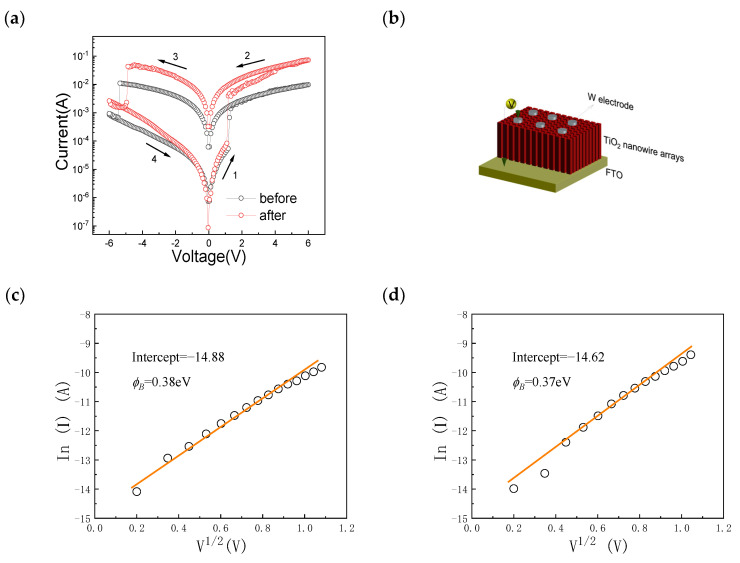
(**a**) The semi-logarithmic *I-V* curves of the W/TiO_2_/FTO memory device before and after annealing. (**b**) Schematic diagram of the W/TiO_2_/FTO memory device. The *I-V* curves plotted in $In\left(I\right)\sim V^{1/2}$ scale (**c**) before and (**d**) after the rapid nitrogen annealing treatment.

The typical current–voltage (*I-V*) measurements of the W/TiO_2_/FTO memory device were carried out to explain the resistive switching characteristics of the W/TiO_2_/FTO memory device before and after the rapid nitrogen annealing treatment, as shown in Figure 3a. The *I-V* curve plotted in semi-logarithmic scale was obtained by setting the applied voltage in a sequence of 0 V → +6 V → 0 V → −6 V → 0 V. Figure 3b displays the schematic diagram of the W/TiO_2_/FTO memory device, which is composed of the top W electrode, the TiO_2_ nanowire arrays, and the bottom FTO electrode. During the measurements, the voltages of the W/TiO_2_/FTO memory device were applied to the W electrode with the FTO electrode grounded. It is clear that the W/TiO_2_/FTO memory device showed nonvolatile bipolar resistive switching characteristics. The pristine resistance state of the W/TiO_2_/FTO memory device was the high resistance state (HRS). When the applied voltage increased from 0 V to +6 V, the W/TiO_2_/FTO memory device switched from the HRS to the LRS with a steep jump of current at +1.17 V (V_set_), which indicated that the set process occurred. After that, the W/TiO_2_/FTO memory device maintained the LRS before the applied voltage reduced to −5.36 V (V_reset_). Subsequently, the reset process occurred at V_reset_, which induced a switch to the pristine HRS with a dramatic decrease in the current in the device, indicating the nonvolatile bipolar resistive switching behavior of the W/TiO_2_/FTO memory device. After the rapid nitrogen annealing treatment, the V_set_ and V_reset_ reduced to 1.09 V and −4.87 V, respectively, and the operation current in the LRS for the device was higher than that of the device before the rapid nitrogen annealing treatment. Figure 3c,d show the *I-V* curves plotted in $In\left(I\right)\sim V^{1/2}$ scale before and after the rapid nitrogen annealing treatment, respectively. It is observed that the W/TiO_2_ Schottky barrier heights of the W/TiO_2_/FTO memory device were about 0.38 eV and 0.37 eV before and after the nitrogen annealing, respectively. Thus, the change in the W/TiO_2_ Schottky barrier modified by the oxygen vacancies is suggested to be responsible for the resistive switching characteristics of the W/TiO_2_/FTO memory device.

The current–voltage characteristics of the Schottky emission are described as [29]:

(1) $$J\propto AT^{2}\exp[\frac{-q\left(\emptyset_{B}-\sqrt{qV/4\pi \varepsilon }\right)}{kT}],$$

where *J* is the current density, *T* is the absolute temperature, *V* is the electric field, A is the Richardson constant, *k* is the Boltzmann’s constant, ε is the dielectric constant, *q* is the electric charge, and $\emptyset_{B}$ is the Schottky barrier height. According to the above fitting results, the conduction behaviors of the W/TiO_2_/FTO memory device are attributed to the Schottky emission in the high resistance state, and the change in the W/TiO_2_ Schottky barrier depletion layer thickness and barrier height modified by the oxygen vacancies at the W/TiO_2_ interface is suggested to be responsible for the resistive switching characteristics of the W/TiO_2_/FTO memory device. For the W/TiO_2_/FTO memory device, the work function of tungsten was 4.6 eV, and the work function of the intrinsic rutile TiO_2_ was about 4.2 eV. Therefore, when the W/TiO_2_ Schottky junction was formed, electrons from the Fermi level of the rutile TiO_2_ migrated toward the W until the Fermi levels on both sides equalized, and the height of the W/TiO_2_ Schottky junction depletion barrier was about 0.4 eV. As shown in Figure 3, the Schottky barrier heights of the W/TiO_2_ Schottky barrier depletion layer were about 0.38 eV and 0.37 eV before and after the rapid nitrogen annealing treatment, respectively, which were smaller than that of the intrinsic rutile TiO_2_ because of the existence of oxygen vacancies in the rutile TiO_2_.

**Figure 4:**
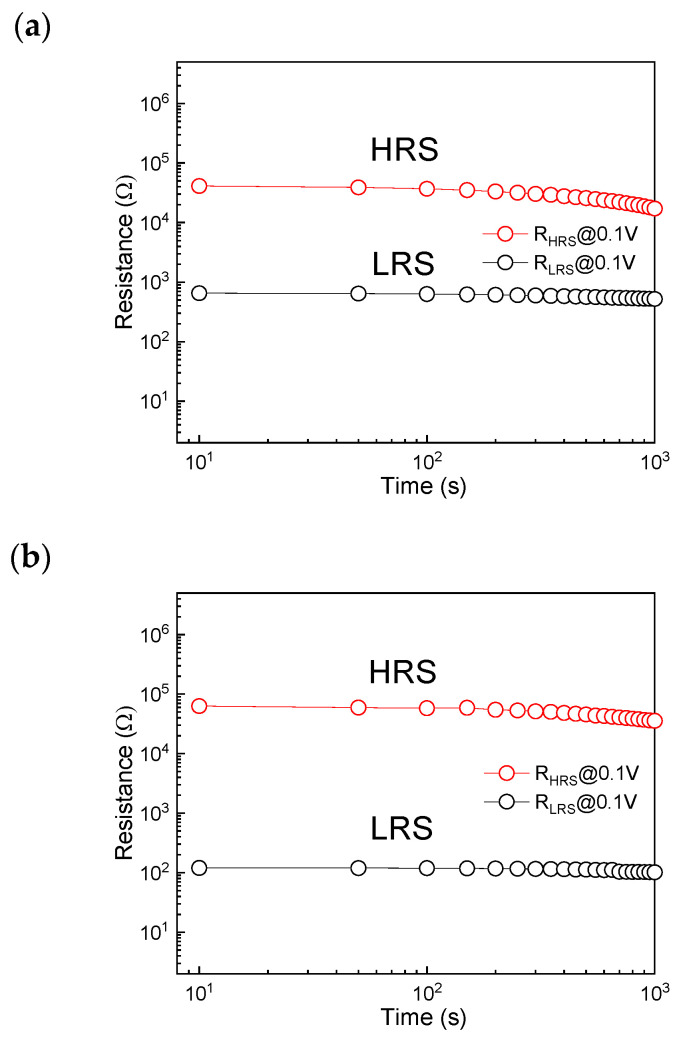
Retention tests of the device at the reading voltage of 0.1 V (**a**) before and (**b**) after the rapid nitrogen annealing treatment.

In order to evaluate the effect of the nitrogen annealing on the resistive switching characteristics of the W/TiO_2_/FTO memory device, Figure 4a,b exhibit the retention tests of the device at the reading voltage of 0.1 V before and after the rapid nitrogen annealing treatment, respectively. As shown in Figure 4a, the W/TiO_2_/FTO memory device displayed the nonvolatile resistive switching characteristics with a high resistance ratio (R_HRS_/R_LRS_) of about two orders of magnitude, which could be stably preserved for over 10^3^ s without obvious degradation. Figure 4b indicates the retention tests of the device at the reading voltage of 0.1 V after the rapid nitrogen annealing treatment, it is appreciable that the R_HRS_/R_LRS_ of the W/TiO_2_/FTO memory device increased from about two orders of magnitude to three orders of magnitude after the rapid nitrogen annealing treatment. In comparison with the previous reports about TiO_2_ memory devices as summarized in Table 1 [16,17,18,19,21,22,23,24,25,26,27,28,29,31], the W/TiO_2_/FTO memory device in this work has a relatively lower V_set_ and the highest resistance ratio of about three orders of magnitude, which demonstrates the outstanding potential of the W/TiO_2_/FTO memory device for the future nonvolatile memory applications.

### 3.4. The Resistive Switching Mechanism of the W/TiO_2_/FTO Memory Device

**Figure 5:**
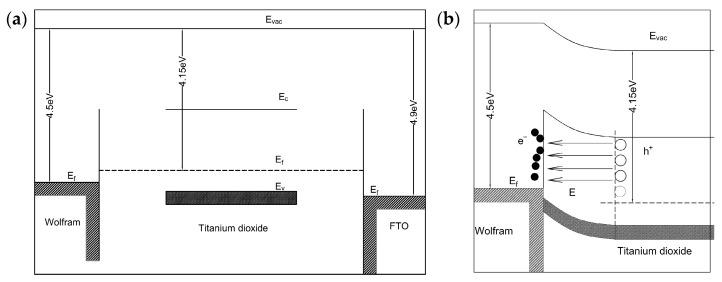
Schematic diagram of the relative band positions of the W, TiO_2_, and FTO (**a**) before and (**b**) after the formation of the W/TiO_2_ Schottky interface.

**Figure 6:**
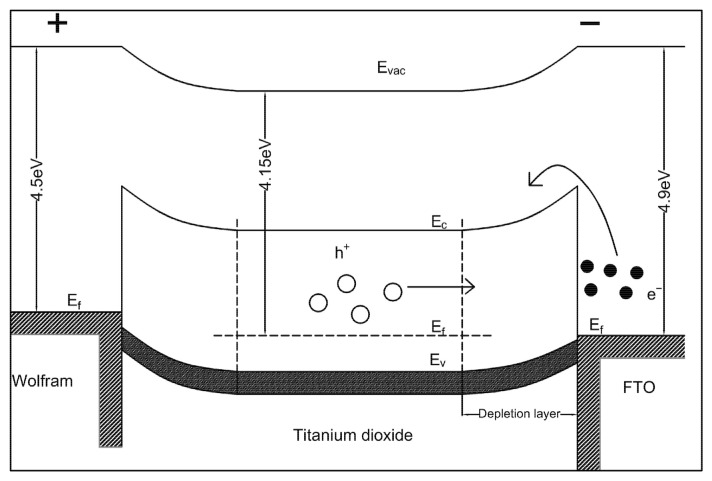
Schematic of the resistive switching mechanism of the W/TiO_2_/FTO memory device.

To further illustrate the resistive switching mechanism of the W/TiO_2_/FTO memory device, Figure 5 shows the schematic diagram of the relative band positions of the W, TiO_2_, and FTO before and after the formation of the W/TiO_2_ Schottky interface. It was found that the work function of W was about 4.6 eV, which was higher than that of TiO_2_ (4.2 eV). Therefore, the electrons migrated from the Fermi level of the TiO_2_ to the W electrode until the Fermi levels on both sides equalized when the W/TiO_2_ Schottky interface was formed, which pushed the conduction band of the W electrode to a relatively higher energy level with respect to the conduction band position of the TiO_2_. Thus, the change in the W/TiO_2_ Schottky barrier depletion layer thickness and barrier height modified by the oxygen vacancies at the W/TiO_2_ interface is suggested to be responsible for the resistive switching characteristics of the W/TiO_2_/FTO memory device. During the set process as displayed in Figure 6, when the positive voltage was applied to the W/TiO_2_/FTO memory device, the electrons were injected from the bottom FTO electrode and captured by the oxygen vacancies in the W/TiO_2_ Schottky barrier depletion layer, resulting in lowering the W/TiO_2_ Schottky barrier height and barrier thickness. Once the W/TiO_2_ Schottky barrier became low and thin enough, large amounts of electrons from the TiO_2_ side crossed the W/TiO_2_ Schottky barrier, thus switching the W/TiO_2_/FTO memory device from the HRS to the LRS with an abrupt increase in the current at V_set_. Subsequently, the device maintained the LRS until a large enough negative voltage V_reset_ was applied, indicating the nonvolatile resistive switching behavior of the W/TiO_2_/FTO memory device. During the reset process, when the negative voltage was applied to the W/TiO_2_/FTO memory device, the electrons injected from the top W electrode were retarded by the W/TiO_2_ Schottky barrier, which induced a large electric field to cross the W/TiO_2_ Schottky barrier depletion layer. Thus, the electrons captured by the oxygen vacancies were activated and emitted from the W/TiO_2_ Schottky barrier depletion layer to the bottom FTO electrode, which induced a recovery to the initial state of the W/TiO_2_ Schottky barrier, and the device switched back from the LRS to the HRS with a drastic drop in the current at V_reset_. This work suggests that the W/TiO_2_/FTO memory device may be a potential candidate for future nonvolatile memory applications.

After the rapid nitrogen annealing treatment, N atoms entered the surface of the TiO_2_ nanowire arrays and replaced the O atoms, which resulted in the increase in oxygen vacancies at the W/TiO_2_ Schottky barrier. It is worth noting that the increase in the oxygen vacancies at the W/TiO_2_ Schottky interface led to reducing the depletion layer thickness and lowering the barrier height of the W/TiO_2_ Schottky barrier. Thus, a lower Schottky barrier height of 0.37 eV and smaller set voltage of 1.09 V (V_set_), as well as a higher resistance ratio of about three orders of magnitude, were observed after the rapid nitrogen annealing treatment, as shown in Figure 3.

## 4. Conclusions

In this paper, the rutile TiO_2_ nanowire-based W/TiO_2_/FTO memory device with a high resistance ratio of about three orders of magnitude was successfully obtained by the rapid N_2_ annealing treatment. The as-prepared W/TiO_2_/FTO memory device exhibits nonvolatile bipolar resistive switching behavior. Furthermore, the R_HRS_/R_LRS_ of the W/TiO_2_/FTO memory device was obviously increased from about two orders of magnitude to three orders of magnitude after the rapid N_2_ annealing treatment. The conduction behaviors of the W/TiO_2_/FTO memory device are attributed to the Ohmic conduction mechanism and the Schottky emission in the low resistance state and the high resistance state, respectively. In addition, the change in the W/TiO_2_ Schottky barrier depletion layer thickness and barrier height modified by the oxygen vacancies at the W/TiO_2_ interface has been suggested to be responsible for the nonvolatile resistive switching phenomena of the W/TiO_2_/FTO memory device. This work demonstrates that the rutile TiO_2_ nanowire-based W/TiO_2_/FTO memory device may be an outstanding candidate for application in the future nonvolatile memory devices.

## Figures and Tables

**Table 1:** Summary of the performance parameters for the TiO_2_ based memory devices.

Device Structure	V_set_/V_reset_ (V)	Preparation Method	R_HRS_/R_LRS_ Ratio	Retention (s)	Reference
Al/TiO_2_/TiO_x_/FTO	~+4/~−4	Hydrothermal	>20	3 × 10^4^	[16]
Pt/TiO_2_ NRAs/FTO	+1.5/−1.5	Hydrothermal	~10	3 × 10^5^	[17]
Al/N-TiO_2_ NARs/FTO	~−3/~+3	Hydrothermal	>16	-	[18]
Ag/[TiO_2_/α-Fe_2_O_3_]/FTO	~+4/~−4	Hydrothermal	~10	10^3^	[19]
Ni/TiO_2_/Cu	−0.5/+0.35	Magnetron sputtering	~10	1.5 × 10^3^	[21]
Ir/TiO_x_/TiN	~−1/~+1.5	Magnetron sputtering	~10	10^4^	[22]
Au/Cu NWs/TiO_2_ NTAs/Ti	+0.6/−0.9	Electrochemical anodization	>40	10^4^	[23]
Ag/TiO_2_/Ti	+0.59/−0.58	Anodic oxidation	~27	-	[24]
Pt/Na-doped TiO_2_/Pt	~+2/~−2	Atomic layer deposition	~30	10^5^	[25]
Pt/TiO_x_/W	−5/+2	Atomic layer deposition	>10	10^4^	[26]
Au/TiO_2_ nanotube/FTO	+2.5/−1.3	Electrochemical deposition	~9	-	[27]
Cu/TiO_2−δ_/Pt	+1.5/−1.5	Pulsed laser deposition	10	5 × 10^3^	[28]
ITO/TiO_2_/FTO	~+1/~−0.8	Chemical solution deposition	>10	10^4^	[29]
Ti/TiO_x_/Pt	~+1.5/~−1.4	Magnetron sputtering	~10	-	[31]
W/TiO_2_/FTO	+1.09/−4.87	Hydrothermal	~10^3^	10^3^	This work

## Data Availability

The review was based on publicly available academic literature databases.
